# Forced intensity-controlled endurance training on a small-animal treadmill machine inducing murine cardiac hypertrophy: insights and comparison to voluntary running models

**DOI:** 10.3389/fphar.2025.1682751

**Published:** 2025-10-09

**Authors:** Maximillian Fischer, Agus Simahendra, Tobias Straub, Stefan Brunner, Bartolo Ferraro, Ludwig T. Weckbach

**Affiliations:** ^1^ Department of Medicine I, LMU University Hospital, LMU Munich, Munich, Germany; ^2^ DZHK (German Center for Cardiovascular Research), Partner Site Munich Heart Alliance, Munich, Germany; ^3^ Core Facility Bioinformatics, Biomedical Center, Ludwig-Maximilians-University Munich, Munich, Germany; ^4^ Biomedical Center, Institute of Cardiovascular Physiology and Pathophysiology, Ludwig-Maximilians-University Munich, Munich, Germany

**Keywords:** cardiomyocytes, physiological cardiac hypertrophy, endurance training, small-animal treadmill, exercice

## Abstract

Endurance training is associated with decreased cardiovascular-related morbidity and mortality. Cardiac hypertrophy is an adaptive mechanism, and murine exercise models for cardiac hypertrophy are still under discussion. Using a small-animal treadmill, a forced intensity-controlled training model was conducted to characterize cardiac hypertrophy in mice utilizing multimodal analyses and then compared to datasets of voluntary running mice. Wild-type male C57BL/6 mice at 8 weeks old were subjected to forced endurance training using a small-animal treadmill or sedentary age-matched control. Five different measurement points (0-, 2-, 4-, 8-, and 12 weeks) were used to assess phenotypic changes. Each training group was scanned using an ECG-gated ^18^F-FDG PET/CT scan to evaluate cardiac volumetric parameters. Morphometric analyses were performed for body, heart, and tibia length. Heart samples were used for staining to measure cross-sectional area, inflammatory cell infiltration, and fibrosis. In addition, transcriptomic analysis of 8-week training hearts was evaluated using RNA sequencing. Endurance training promotes significant body weight loss in training mice as early as 2 weeks. After 8 and 12 weeks of training, the heart weight/tibia length ratio was significantly higher than the control. Cardiomyocyte (CM) cross-sectional areas were enlarged by 1.8-fold and shifted to the increased surface area upon training. The CM size plateaued after 8 weeks of forced training. No accompanying inflammation or fibrosis in the training heart was detected, confirming a physiological hypertrophic response induced by forced endurance training. RNA sequencing revealed several genes involved in the cell cycle, apoptosis, contractile protein expression, and organ growth that were among the most differentially regulated genes after 8 weeks of exercise. Forced running showed a more robust gene expression than the published voluntary running model, focusing on growth, hypertrophy, and insulin-like growth factor-related genes. This study investigated the morphometric, histologic, functional, and transcriptomic alterations in cardiac hypertrophy induced by forced intensity-controlled treadmill exercise and discusses its advantages compared to voluntary running models.

## 1 Introduction

Cardiovascular and degenerative diseases are the primary cause of morbidity and mortality in industrialized nations and developing countries (Ponikowski et al., 2016; Cosentino et al., 2020; Knuuti et al., 2020). Exercise training has been established as one of the most important cornerstones to increase cardiorespiratory fitness and reduce major cardiovascular adverse events (Ruegsegger and Booth, 2018).

Physiological heart hypertrophy also occurs in pregnancy, but as opposed to exercise training, hearts in pregnant women adapt mainly to the increasing preload due to expanding blood volume to provide nutrients to the developing fetus (Mone et al., 1996; Dorn et al., 2007). Besides that, hereditary cardiac hypertrophy, such as familial hypertrophic cardiomyopathy, is a classic example that affects young patients. Although enlarged cardiomyocytes (CM) characterize physiological cardiac hypertrophy and pathological familial hypertrophic cardiomyopathy, the disease state is characterized by concentric cardiac hypertrophy with asymmetrical left ventricular hypertrophy predominantly of the interventricular septum. Disease manifestations encompass various clinical manifestations, including heart failure, malignant arrhythmia, and sudden cardiac death (Maillet et al., 2013; Van Berlo et al., 2013).

Furthermore, pathologically enlarged CMs associated with interstitial fibrosis and myocyte disarray occur due to genetic mutations, storage disease, mitochondrial disease, and triple repeat syndromes (Morita et al., 2008). Several pathological conditions, such as chronic arterial hypertension and valvular heart diseases, produce maladaptive cardiac enlargement, increased cardiac stiffness, and augmentation of left ventricular wall thickness without a compensatory increase in left ventricular end-diastolic diameter, known as concentric hypertrophy. Moreover, chronic concentric cardiac hypertrophy could lead to eccentric hypertrophy with hallmarks of chamber dilatation, mitochondrial dysfunction, and cell death if the source of pathological stimuli is not managed appropriately (Nakamura and Sadoshima, 2018). Pathological cardiac hypertrophy is associated with an inflammatory process, programmed cell death, inefficient calcium utilization, rarefaction of vascular density, interstitial fibrosis, CM disarray, and systolic and diastolic heart failure (Shimizu and Minamino, 2016; Nakamura and Sadoshima, 2018). Concentric hypertrophy exhibited a pronounced increase in the wall and interventricular septum thickness, reducing the left ventricular cavity. CM thickness and width rise without compensatory muscle fiber elongation and can be histologically observed in CM specimens with pathological hypertrophy caused by increased afterload *in vitro* (Hirt et al., 2012).

Distinctive signaling cascades drive the differentiation between physiological and pathological cardiac hypertrophy. Moreover, alterations of various molecular processes, such as cellular metabolism switching to glucose, energy substrate utilization, excessive reactive oxygen species (ROS) production, inflammation, gene expressions, protein translation, and sarcomere organization, arise, leading to the progression of adverse cardiac remodeling (Gibb and Hill, 2018).

Regular exercise has been proclaimed as a significant protective measure to reduce cardiovascular ischemia, injury, and mortality (Joyner and Green, 2009). Unfortunately, these physiological changes could also be chronically overwhelmed and lead to maladaptive responses in certain pathological conditions, such as decompensated heart failure or ischemic heart disease, when excessive neuro-humoral, inflammatory, and remodeling responses take place (Diuresis Jönsson et al., 2014; Bayes-Genis et al., 2016; Hartupee and Mann, 2016).

Due to exercise, hearts undergo adaptive modifications in their mass, volume, and metabolism. Aerobic exercise training leads to numerous hemodynamic adaptive changes when performed regularly and chronically. These can be translated roughly to increased cardiac performance and described as exercise-induced cardiac remodeling (Pal et al., 2013). The enlargement of the cardiac dimension can be easily detected by clinical imaging techniques (e.g., echocardiography or cardiac magnetic resonance imaging (MRI)), showing an increase in several cardiac parameters (Sharma et al., 2015).

Endurance training is associated with a lower incidence of cardiovascular-related diseases. The intensity and frequency of aerobic exercise training to induce these desirable positive effects remain under discussion. In addition, there have been scientific reports suggesting that vigorous endurance exercise training in rodents might promote cardiac collagen deposition, fibrosis marker elevation, deleterious electrical remodeling, and create a substrate for cardiac arrhythmias, particularly atrial fibrillation and ventricular tachycardia (Benito et al., 2011). Moreover, adaptive structural and functional remodeling is expected to occur in training athletes after several weeks of moderately intense regular endurance training. Nevertheless, the exact time point, training intensity, and additional scientific data on myocardial performance and possible deleterious effects of intense exercise remain to be elucidated (Eijsvogels et al., 2016). Due to the limitations of study approaches in humans, animal models are available to understand better the biological processes involved in physiological hypertrophy. Several experimental strategies have been developed to mimic human endurance training, such as forced running exercises using small-animal treadmill machines. Voluntary wheel running and swimming exercises with specific training protocols were also widely used in mice and rats.

Animals can provide valuable information depicting underlying mechanisms of cardiovascular physiological changes during physical exertion and their phenotypic consequences, including molecular analyses in healthy heart tissue, which are almost impossible to perform in humans due to ethical reasons. Endurance training using commercially available treadmill machines encompasses a forced and intensity-controlled exercise. It also allows adding velocity, training duration, and inclination to control exercise performance, which may generate hypertrophied hearts (Kemi et al., 2002; Hastings et al., 2022). On the other hand, the feasibility and detailed training programs have culminated in a tremendous amount of controversy (Wang et al., 2010).

We show that forced and intensity-controlled endurance training using a treadmill machine leads to the development of cardiac hypertrophy and transcriptomic alterations in the heart. The study objective was the multimodal analysis of exercise-induced cardiac remodeling in mice to evaluate morphometric parameters, cardiac volume, and function, and especially explore the altered molecular signaling pathway by RNA sequencing compared to sedentary controls and the model of voluntary running, which has not been published before.

## 2 Materials and methods

### 2.1 Mice

C57BL/6 mice aged 8- weeks old with a weight of 23.04 g ± 2.27 were purchased from Charles River (Sulzfeld, Germany). All animal studies were performed following the European Directive 63/2010/EU guideline and extrapolated to Animal Protection Law in the scope of German legislation. The animals were housed in a controlled environment with free access to water and a standard chow diet. Our study protocols were reviewed and approved by the governmental animal ethics committee and the local government (ROB-55.2-2532.Vet_02-20-166). Animal health and stress monitoring during forced exercise was conduced and controlled by experienced researchers and vets according to score sheets approved by the local goverment. Measures to minimize stress, where additional enviromental enrichment (e.g.,: nesting material), larger cages in combination with adequate acclimatization periods.

### 2.2 Treadmill exercise protocol

Wild-type mice were randomly assigned to forced endurance training or sedentary counterparts. Forced exercise training was performed using a dedicated small-animal treadmill (Panlab Harvard Apparatus, Holliston, Massachusetts). The animals rested for at least 2 weeks before the training protocol to avoid unnecessary stress and an additional week of acclimatization. The acclimatization started with a speed of 10 m/min for 10 min on the first day. The speed and duration of training were incrementally increased each day until the fifth day, namely 11 m/min, 12 m/min, 13 m/min, and 14 m/min for 20, 30, 40, and 50 min respectively. After 1 week of acclimatization, the training protocol started with the speed of 15 m/min for 60 min every day at the same time, 5 days a week for several weeks (0 until 4 weeks), depending on their allocated groups. The 2- and 4-week groups were allocated randomly to run for 60 min/day. The 8- and 12-week groups ran for 120 min/day. 10 animals ran for 2, 4, and 8 weeks each, and 5 animals trained for 12 weeks. The exact number of mice were allocated as sedentary controls in each time frame. For baseline evaluation, 10 animals were trained for only 1 week using an acclimatization protocol, designed as a 0-week training group, and compared with their respective sedentary control. Before each training frame, mice were adapted to the treadmill environment for 15 min. They are allowed to rest every 15 min for 2 min during each training session.

### 2.3 Morphometric phenotyping

The morphometric analysis was performed after each PET/CT scan at the end of the planned training schedule. Hearts were harvested, washed in cold PBS, and weighed using Pioneer Ohaus analytical balance (Sigma-Aldrich, Steinheim, Germany) and processed in 4% formaldehyde solution (Sigma-Aldrich, Darmstadt, Germany) for histological processing. The right tibia length (mm) was measured using Preciva^®^ digital caliper BellaCocool GmbH (Berlin, Germany).

### 2.4 Immunochemistry and histology

Hearts were fixated in 4% formaldehyde solution for 4–24 h, followed by incubation in 30% sucrose solution (Sigma-Aldrich, Steinheim, Germany) overnight for dehydration. The hearts were cut transversally at the height of ventricular papillary muscle and embedded in Tissue-Tek OCT mounting medium (Sakura Finetek, Alphen aan den Rijn, Netherlands) inside the Tissue-Tek Cryomold standard size of 25 × 20 × 5 mm (Sakura Finetek, Alphen aan den Rijn, Netherlands). Specimens were then stored in a −20 °C fridge overnight or at −80 °C for long-term storage. Frozen hearts were further cut using cryotome Leica CM3050 S (Leica Biosystems, Wetzlar, Germany) with a thickness of 8 µm.

#### 2.4.1 Wheat germ agglutinin (WGA) immunofluorescence staining

The slides with heart tissue sections were warmed at room temperature for 10 min, followed by refixation in 4% formaldehyde solution for 10 min. The fixated sections were washed thrice for 5 min each with PBS-Tween 20^®^ 0.1% (Sigma-Aldrich, Steinheim, Germany). The specimens’ glasses were made dry at room temperature for 10–15 min, and the desired staining area was encircled using a PAP Pen for immunostaining (Sigma-Aldrich, Schnelldorf, Germany). The specimens were applied 50 µL blocking solution [PBS with 0.5% saponin, 1% BSA, and 10% goat serum (Sigma-Aldrich, Steinheim, Germany)]. After 1 hour of incubation, the blocking solution was washed off three times using Hanks’ Balanced Salts Solution/HBSS (Biowest, Nuaillé, France). Wheat germ agglutinin (WGA) conjugated with Alexa Fluor^®^ 647 (Invitrogen, Eugene, United States, cat. W32466) staining solution was diluted 1: 100 in HBSS and poured onto the specimens for 1.5 h incubation at room temperature inside a dark, humid incubation chamber (Simport Scientific, Beloeil, Canada). The incubation was followed by washing twice with HBSS. After the washing steps, SYTOX^®^ green dye (Invitrogen, Eugene, United States, cat. S7020) diluted 1: 1000 in HBSS was applied to the specimens, followed by 10 min of incubation inside the dark and humid chamber at room temperature. The specimens were again soaked with HBSS three times and kept dry at room temperature for 10–15 min. Subsequently, the sections were covered with ProLong^®^ Gold anti-fade reagent (Invitrogen, Eugene, United States) as mounting media, sealed with coverslips size 24 × 50 mm (R. Langenbrinck GmbH, Emmendingen, Germany), and dried at room temperature for 24 h in the dark before microscopical analysis.

#### 2.4.2 Hematoxylin-eosin staining

The specimens’ glasses were warmed at room temperature for 10 min and washed in Aqua (B-Braun, Melsungen, Germany) and PBS for 10 and 5 min to remove the OCT mounting media. The specimens were then put into Meyer’s Hematoxylin Solution (Carl Roth, Karlsruhe, Germany) for 3–5 min, followed by washing steps under the flowing tap water for 15 min. The specimens were then reconditioned in Aqua solution for 2 min and dipped into Eosin G Solution 0.5% (Carl Roth, Karlsruhe, Germany) mixed with one drop of acetic acid glacial (Merck, Darmstadt, Germany) as buffer and preservatives for 3 min. The stained specimens were washed once using distilled water for 2 s, followed by dehydration steps in the sequence of incrementally concentrated alcohol solution 70%, 96%, and 100% (Sigma-Aldrich, Steinheim, Germany) for 5 min each. At last, the specimens were put into Xylene I and II solution (Sigma-Aldrich, Steinheim, Germany) for 5 min each, dried at room temperature, mounted in Roti^®^ Histokitt II (Carl Roth, Karlsruhe, Germany), and covered with coverslips size 24 × 50 mm before analysis under the microscope.

#### 2.4.3 Picrosirius red staining

After warming up for 10 min at room temperature, the heart specimens were washed in PBS for 5 min, followed by staining in Direct Red 80 (Sirius Red) 0.1% solution (Sigma-Aldrich, Steinheim, Germany) for 1 h. The stained samples were then washed in 0.01 N hydrochloric acid (Merck, Darmstadt, Germany) for 2 min, followed by serial dipping into a sequence of a concentrated alcohol solution of 50%, 70%, 96%, and 100% for dehydration purposes. Finally, the dehydrated stained specimens were immersed in Xylene I and II solution for 2 min each, dried at room temperature, mounted in Roti^®^ Histokitt II, and covered with coverslips size 24 × 50 mm before analysis under the microscope.

### 2.5 Microscopical image acquisition and analysis

A confocal microscope Leica TCS SP8 X (Leica Microsystems, Wetzlar, Germany) was used to image the cross-sectional area of transversally cut heart fibers at ×40 magnification, respectively. The Type-F Immersion Liquid (Leica Microsystems, Wetzlar, Germany) was applied to each image acquisition for better object visualization. The images were taken utilizing 488 nm to detect SYTOX^®^ green and 633 nm to visualize WGA. The heart samples were analyzed at the height of the papillary muscles on the left ventricle. The images were made in triplicate, and the surface area analysis was performed blinded using ImageJ2 (NIH, Maryland, USA). The quantification of both heart surface areas was performed manually on a one-to-one section basis (n > 200 cells per section, 600–800 cells per heart sample, and n > 100 per section), and the triplicate results were averaged. A stereo microscope Leica M205 FA (Leica Microsystems, Wetzlar, Germany) was utilized to visualize stained specimens with Hematoxylin-Eosin and Picrosirius Red. Images with corresponding magnification for heart specimens were taken in triplicates with a Leica DFC7000 T camera and analyzed using ImageJ2 software to detect inflammatory cell infiltration and fibrosis.

### 2.6 *In vivo* cardiac PET/CT image acquisition

The induction and maintenance of anesthesia were performed using 2.5% and 1.5% isoflurane, respectively. The oxygen flow was maintained at a 1.0–1.4 L/min rate throughout the scan and delivered using a closed tubing system with a face mask. ^18^F-FDG was administrated intravenously into the lateral superficial tail veins using a catheter and a volume of 200 μL, resulting in approximately 20 MBq radioactivity. After tracer administration, the catheter was flushed using an isotonic saline solution of 0.9% (Fresenius Kabi, Bad Homburg, Germany) in a volume of 50 µL. A 30-min delay time from injection until the start of scanning enabled radiotracer distribution into the myocardium. Then ECG-gated micro-PET image acquisitions using a Nanoscan^®^ dedicated small animal PET scanner (Mediso Medical Imaging Systems, Budapest, Hungary) were accomplished on 0, 2, 4, 8, 12 weeks groups on each animal, both training and sedentary control. A heating pad and a rectal thermometer for tight temperature monitoring were placed under and in the prone-positioned animals, respectively, to regulate average core body temperature. The animals remained anesthetized, and the ECG activity was carefully recorded in real-time throughout the entire 30-min scan duration using modified Kendall neonatal ECG electrodes (Cardinal Health, Norderstedt, Germany), which were placed on animal’s both forepaws and left hind paw. The animals’ eyes were protected using Bephantene^®^ cream to avoid dryness during the anesthesia. After image acquisition, a veterinarian observed the animals closely until they fully recovered from the anesthesia. The recorded data were further reconstructed, analyzed, and quantified using Nucline NanoScan 3.04.018.0000 (Mediso Medical Imaging System, Budapest, Hungary), Inveon Research Workplace 4.2 (Siemens Medical Solutions, Malvern, PA, USA), and QPS/QGS^®^ Software (Cedar-Sinai Medical Centers, Beverly Hills, CA, USA) respectively.

### 2.7 PET/CT image analysis

The acquired images were reconstructed as ECG-gated sequences using Nucline NanoScan 3.04.018.0000 (Mediso Medical Imaging System, Budapest, Hungary). ECG-gated image reconstruction was performed using a built-in Tera-Tomo 3D reconstruction algorithm with normal regularization, application median filter, spike filter, edge artifact reduction, 8 iterations, 0.50 voxels size, and subsets number of 6, using 16 bin frames was also normalized, corrected for randoms, dead-, decay time, attenuation, and scatter. The reconstructed images were then exported utilizing InterView FUSION 3.09.008.0000 (Mediso Medical Imaging System, Budapest, Hungary) for further processing. Exported PET images were then analyzed using the Inveon Research Workplace 4.2 (Siemens Medical Solutions, Malvern, PA, United States) (Fischer et al., 2021). The heart rate records during the scan were extracted from the automatically saved log data and used to verify the accuracy of the ECG-triggering signal. Gated images were then cropped and rescaled by augmenting the values to the factor of 10 using the R Program for Statistical Computing (Lucent Technologies, Murray Hill, NJ, USA) for further analysis. Gated images are smoothened using Gaussian σ 0.75 algorithms in the x, y, and z-axis. The three-dimensional left ventricular function parameters (EDV, ESV, SV, and EF) were quantified utilizing QGS^®^ (Cedars-Sinai, Los Angeles, CA, United States) on the ECG-gated reconstruction images. Subsequently, the cardiac output (µL/min) was calculated by multiplying the quantified SV with the recorded average heart rate/minute.

### 2.8 RNA isolation and bulk RNA-Seq analysis

The specimens used for RNA isolation were taken from the mice’s whole heart, which consisted of both atriums (including the left and right atrial appendages) and ventricles. The animal’s hearts were soaked directly after extraction into Trizol^®^ reagent (Life Technologies, Carlsbad, USA) (1 mL Trizol^®^ reagent was used pro 100 mg heart tissue) and homogenized using IKA T10 Ultra-Turrax basic Homogenizer (Janke and Kunkel KG, Staufen i. Breisgau). The chloroform solution (Sigma-Aldrich, Steinheim, Germany) was added to the homogenized tissues with 0.2 mL pro milliliter Trizol. The mixture was agitated for 15 s and incubated at room temperature for 2–3 min. The samples were centrifuged at 13,000 rpm at 4 °C for 15 min to separate the upper aqueous-, lower emulsion- and middle interphase. The upper aqueous phase containing the desired RNA was transferred into new clean tubes. The isolated RNA was washed by agitation for 15 s with 500 µL Isopropanol 99.7% (Sigma-Aldrich, Steinheim, Germany), each 1 mL sample followed by 10 min incubation at room temperature. After centrifugation, the pellets containing the precipitated RNA were afterward cleansed with 1 mL 75% ethanol followed by centrifugation at 10,000 rpm at 4 °C for 8 min. The supernatant was discharged, and the isolated pellet containing the desired RNA was dried at room temperature before adding 30 µL RNA-free water for dilution. A Nanodrop 2000 photometer (Thermo Fisher, Waltham, United States) was used to determine the quantity and quality of the isolated RNA. High-quality total RNA samples have an A260/280 ratio ≥1.9 indicating the absence of contaminating substances.

A total of 10 samples of five 8 weeks of training and five sedentary mice at the same age were analyzed by bulk RNA-sequencing. The RNA sequencing libraries were generated with NEBNext^®^ Ultra™ II Directional RNA Library Prep technology (New England Biolabs, Ipswich, United States) according to the manufacturer’s instructions and internal laboratory quality control. The library generation uses fragmentation, a poly-T oligo, and sequencing adapter ligation. One RNA sequencing library pool was prepared from the 10 generated RNA-Seq libraries. The template amplification and clustering were performed using the Illumina NovaSeq^®^ 6000 next-generation sequencing system applying the exclusion amplification chemistry (Illumina, San Diego, United States), and its high output mode was made with 1 × 100 bp single-read chemistry. The cluster generation and RNA sequencing were operated under the control of the NovaSeq Control Software (NVCS) v1.6.0 (Illumina, San Diego, United States). The processing of primary images was performed on the NovaSeq instrument using Real-Time Analysis (RTA) 3.4.4 software (Illumina, San Diego, United States). Datasets for the voluntary running model were deposited and publicly available under the following link: https://www.ncbi.nlm.nih.gov/sra/?term=PRJNA811435 (Lerchenmüller et al., 2022)

The evaluation of the imaging and sequencing running performance utilized the Illumina Sequence Analysis Viewer (SAV) v2.4.7 (Illumina, San Diego, United States). Sequencing reads were aligned to the mouse reference genome (version GRCm39.107) with STAR (version 2.7.10a). Expression values (TPM) were calculated with RSEM (version 1.3.3). Post-processing was performed in R/bioconductor (version 4.2.0) using default parameters if not indicated otherwise. Differential gene expression analysis was performed using DEseq2 (version 1.36.0). An adjusted p-value (FDR) of less than 0.1 was used to classify significantly changed expression. Gene set enrichment analyses were conducted with package ‘fgsea’ (version 1.22.0) using the GO gene sets (biological process only) of ‘msigdbr’ (version 7.5.1). Genes were ranked based on the DEseq2 test statistic. The analysis was limited to gene sets ranging from 15 to 500 in size.

### 2.9 Statistical analysis

Statistical analyses were performed using GraphPad Prism (Version 8, Inc., San Diego, California). All results were shown as mean with standard deviation. One-way and two-way ANOVA analyses, including multiple comparisons and paired and unpaired student t-tests, were performed where applicable for normally distributed data sets. The Wilcoxon sign-rank, Kruskall-Wallis, or Mann-Whitney U-test was used for groups that were not normally distributed. Differences were considered significant when P < 0.05 (*), <0.01 (**), or <0.001 (***).

## 3 Results

### 3.1 Endurance training induces body weight loss and heart hypertrophy in mice

**FIGURE 1:**
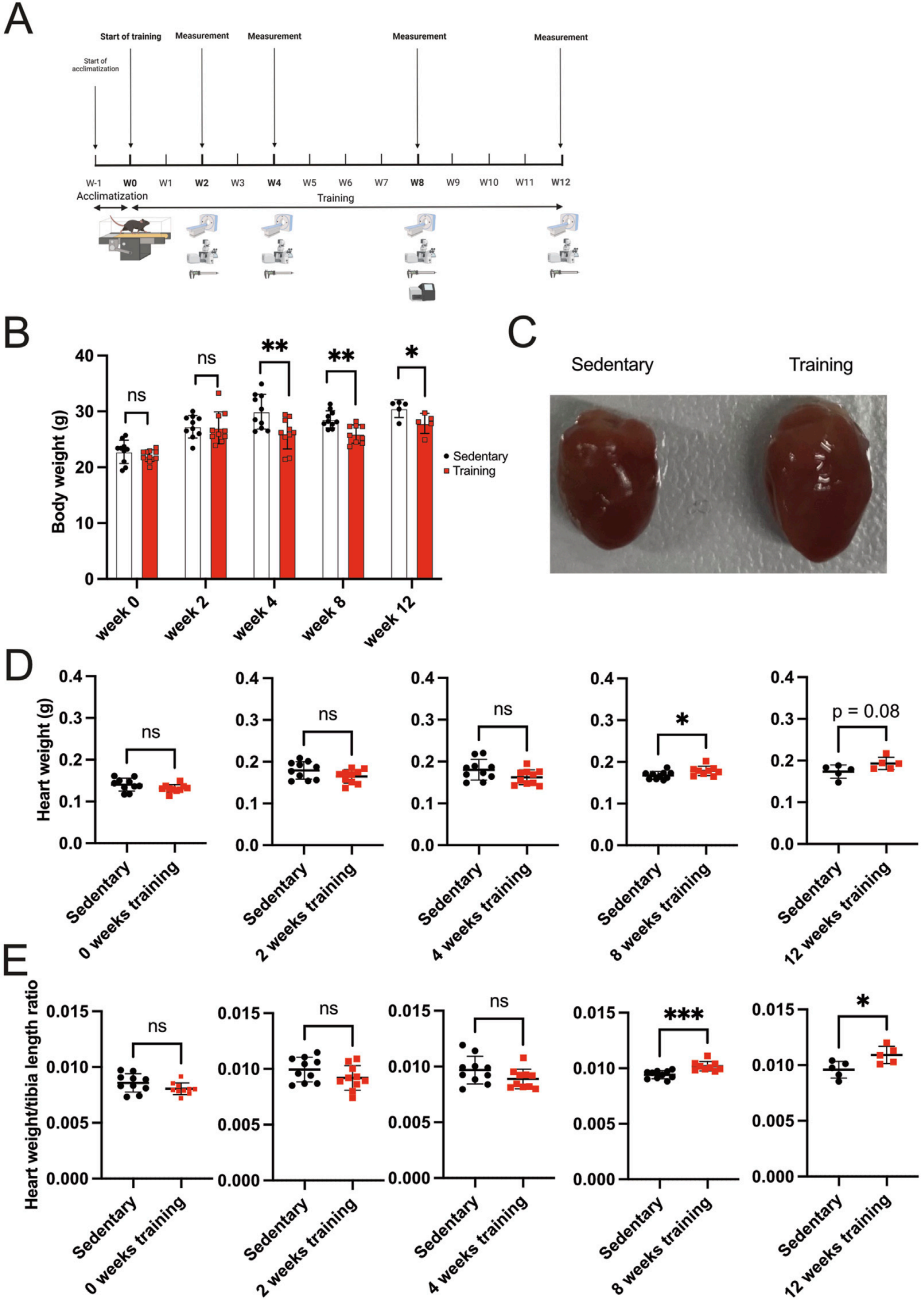
Study design and morphometric analysis of sedentary vs training mice. **(A)** Schematic showing the treadmill running protocol and study design. The scheme includes acclimatization before the training protocol. At week 0 (W0), week 2 (W2), week 4 (W4), week 8 (W8), and week 12 (W12), the training and sedentary mice were sacrificed and analyzed, including ECG-gated PET/CT, histology, morphometrics, and RNA sequencing. **(B)** Mice body weight was recorded on the corresponding weeks (0, 2, 4, 8, and 12). Sedentary control mice served as controls. Data illustrated as mean ± SD. N = 10 mice in each group, except for 12 weeks (n = 5 mice per group). Black dots represent sedentary control; training animals are shown in red. Two-way ANOVA with Turkey’s multiple comparison tests was used. ns = not significant, *p < 0.05, **p < 0.01, ***p < 0.001. **(C)** Representative gross heart in an 8-week training group (right-hand side) and their matched sedentary control (left-hand side). The extraction was performed carefully to include all four heart chambers and exclude pericardial tissue. **(D)** Heart weight on 0-, 2-, 4-, 8-, and 12-week training animals and their matched sedentary control. Data illustrated as mean ± SD. N = 10 mice in each group, except for 12 weeks (n = 5 mice per group). Black dots represent sedentary control; training animals are shown in red. Unpaired two-tailed Student’s t-test was used. ns = not significant, *p < 0.05, **p < 0.01, ***p < 0.001. **(E)** Heart weight/tibia length ratio (g/mm) across different training time points (0, 2, 4, 8, and 12 weeks). Data illustrated as mean ± SD. N = 10 mice in each group, except for 12 weeks (n = 5 mice per group). Black dots represent sedentary control; training animals are shown in red. Unpaired two-tailed Student’s t-test was used. ns = not significant, *p < 0.05, **p < 0.01, ***p < 0.001.

Figure 1A illustrates the study design, depicting different training groups at 2 weeks, 4 weeks, 8 weeks, and 12 weeks. At each time point, mice were analyzed for *in vivo* measurement of cardiac volumes and function by ECG-gated PET/CT. Morphometrics (body weight, heart weight, and tibia length) and histology were performed for each group. RNA sequencing of hearts was performed after 8 weeks of training and in the sedentary control group.

The body weight in the fourth week of training was significantly lower in training compared to sedentary control mice (Figure 1B). The mean body weight of the 4-week training group is 24.65 g ± 2.71 compared to 28.55 g ± 2.47 in their sedentary counterpart (p < 0.01). Similar results were recorded after 8- and 12-week training. The mean average body weight on 8-week training was 25.96 g ± 1.63 in comparison to 28.59 g ± 1.53 in the sedentary group (p < 0.01) as well as 27.84 g ± 1.80 in the 12-week training group as opposed to 30.50 g ± 1.58 on the 12-week sedentary groups (p < 0.05).

To determine the effect of endurance training on heart morphology, we analyzed the hearts of different training groups. Interestingly, after 8 weeks, we found an increased heart mass in training mice compared to the sedentary controls. A representative image of the gross morphology of a mouse’s heart after 8 weeks of training is presented in Figure 1C. Figure 1D depicts the results of heart weight measurement between groups at different training time points. The 8-week training group showed an increased heart weight, indicating a response to the training compared to their matched control (p < 0.05). A strong trend was also observed in the 12-week training group (p = 0.08) compared to their matched 12-week sedentary control. No endurance training effects on the cardiac mass were observed in 2- and 4-week training groups. To avoid any confounders, we analyzed the heart weight/tibia length ratio as a valid indicator of cardiac hypertrophy (Figure 1E). A significant difference in heart weight/tibia length ratio in 8-week training mice compared to the control was evident (p < 0.001). The F-test for equality of variances between the 4-week and 8-week training groups was not significant (F-test: p = 0.5). Using the F-test in ANOVA analysis of 4- and 8-week sedentary and training groups showed the same result, arguing against an influence of low variability in the 8-week groups (F-test: p = 0.1).

Furthermore, the heart weight/tibia length ratio is significantly higher in 12-week training animals compared to their sedentary control (p < 0.05). This finding also confirms our hypothesis that a continuous increase in cardiac mass was appreciated as a response to forced chronic endurance training on the treadmill machine in experimental animals, starting to be observable from 8 weeks as a critical time-point.

### 3.2 Cardiac function analysis derived from small animal PET/CT

**FIGURE 2:**
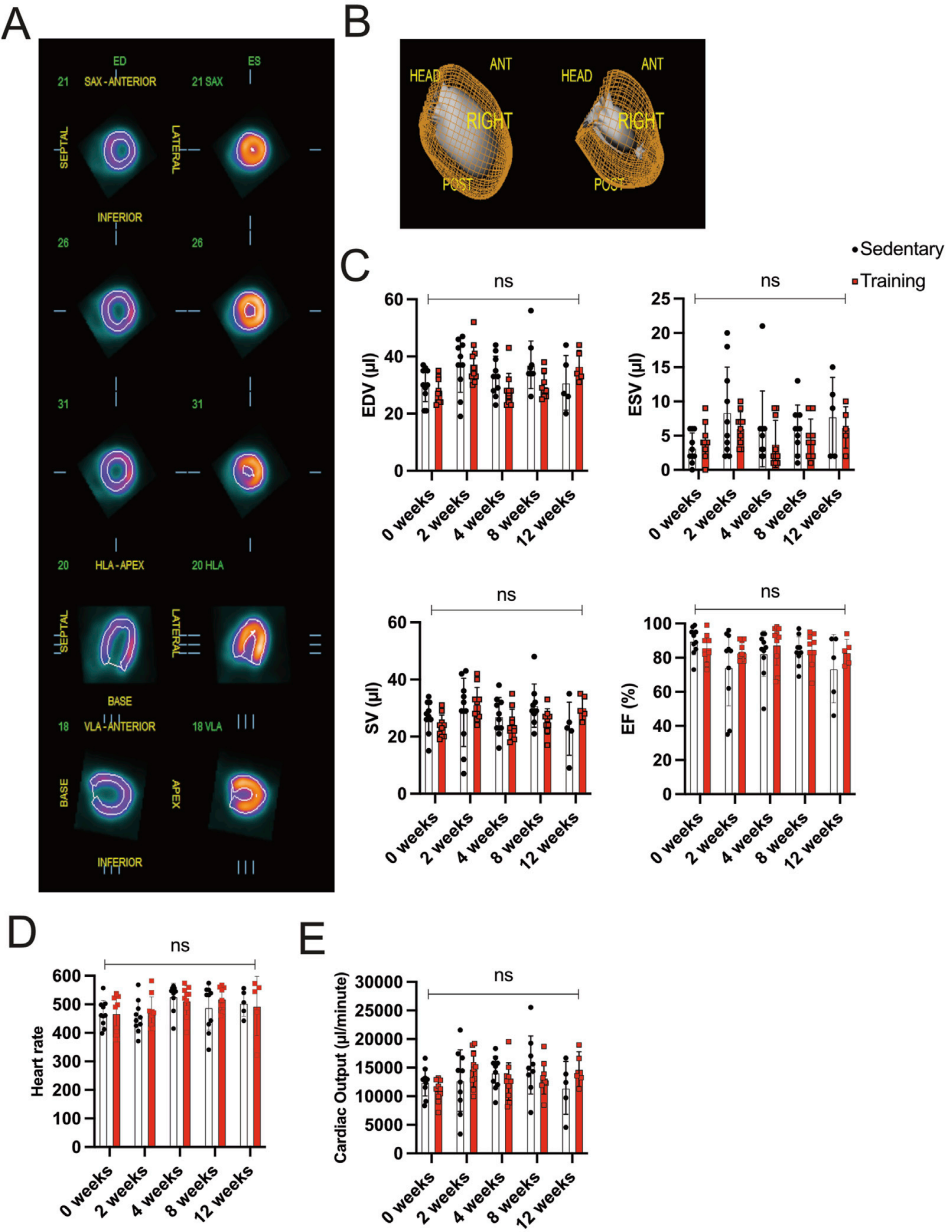
Gated PET/CT assessment of cardiac volume and function **(A)** Representatives of different views (coronal, horizontal long axis (HLA), and vertical long axis (VLA)) of the left ventricle during end-diastole (ED; left side) and end-systole (ES; right side). **(B)** Three-dimensional reconstruction of ECG-gated PET/CT images showing the left ventricle in end-diastole (left) and end-systole (right). **(C)** Evaluation of EDV, ESV, SV, and EF between training and sedentary groups at different time points. **(D)** Heart rate/minute in training animals compared to their matched sedentary controls. **(E)** Cardiac output is calculated from SV and heart rate/minute. Data illustrated as mean ± SD. N = 10 mice in each group, except for 12 weeks (n = 5 mice per group). Black dots represent sedentary control; training animals are shown in red. Two-way ANOVA with Turkey’s multiple comparison tests was used. ns = not significant, *p < 0.05, **p < 0.01, ***p < 0.001.

In this study, the endurance training of 8 weeks promoted cardiac hypertrophy based on morphometric analysis. Further, we explored whether forced chronic endurance training influences left ventricular (LV) volume and function. In our animal experiment, we performed a small animal-dedicated PET/CT image acquisition to assess LV volumes. Figure 2A illustrates the left ventricular ^18^F-FDG uptake in three axes (coronal, horizontal long, and vertical long axis) at end-diastole and end-systole. The analysis is performed using the commercially available QGS software to enable ECG-gated function analysis from PET data from 3D reconstructed images, as illustrated in Figure 2B.

Functional cardiac analysis on training hearts at different time points is illustrated in Figure 2C. No significant difference in EDV, ESV, and SV between sedentary and training animals in the corresponding experimental groups could be detected. The EF of the 8-week training group remained stable compared to the sedentary group (training: 85.00% ± 9.64 vs sedentary 83.78% ± 8.61, p = 0.78). Similar parameter relationships were appreciated in the 2-, 4-, and 12-week groups without showing significance in the corresponding statistical analysis. Furthermore, the SV multiplied the acquired heart rate/minute means to calculate cardiac output (CO), expressed in µl/minute. Figures 2D,E show the data on heart rate/minute and CO across different time points. The difference in heart rate/minute and CO showed no statistical significance in the running groups.

### 3.3 The CM size shifts to greater surface areas after forced endurance training

**FIGURE 3:**
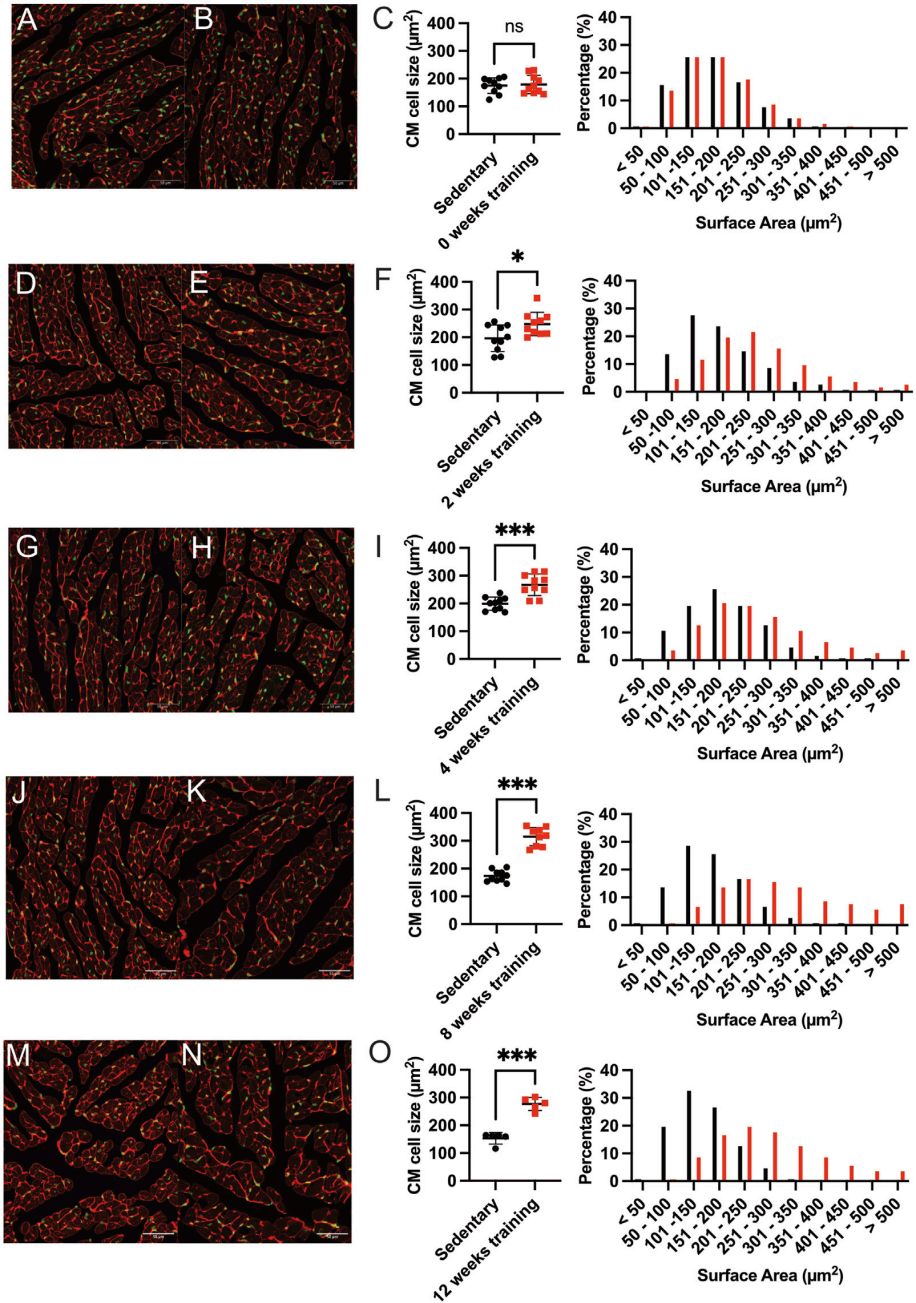
Representative immunofluorescence staining and quantification of induced cardiac hypertrophy. Immunofluorescence images at baseline 0-weeks sedentary control **(A)** and corresponding training group **(B)** WGA-Alexa Fluor^®^ 647 (shown in red) and SYTOX green^®^ (shown in green). The length bar represents 50 µm. **(C)** Quantification of cardiac hypertrophy by cross-sectional CM size (left) and the corresponding percentage of surface area (right) compared with the sedentary controls. Black dots indicated sedentary control mice. Training mice are depicted in red. **(D,E)** show representative immunofluorescence images after 2 weeks of training and the corresponding quantification illustrated in **(F)**. The 4-week training group is shown in **(G–I)**, and the 8-week training group in **(J–L)**. The 12-week training groups in **(M–O)**. Data illustrated as mean ± SD. N = 10 mice in each group, except for 12 weeks (n = 5 mice per group). Unpaired two-tailed Student’s t-test was used. ns = not significant, *p < 0.05, **p < 0.01, ***p < 0.001.

Our phenotypic characterization of different training protocols revealed an increased heart weight/tibia length ratio after 8 weeks of forced exercise training on the treadmill. We, therefore, examine these changes further by histology. We quantified the CM cross-sectional surface area as a reliable method to evaluate cardiac hypertrophy. Figure 3 shows the representative immunofluorescence images comparing the cross-sectional area of CM in the middle cardiac section at the height of the papillary muscles. The CMs were then counted based on the corresponding numbers of SYTOX-green stained nuclei, and the circumferential WGA-stained cell membrane was calculated. Surface areas were tabulated, and the training and sedentary control groups were compared. Furthermore, the percentage of cell numbers in corresponding categories of surface area size represented better insight regarding the cell size distribution. Figures 3A–C show that there was no significant difference observed in mean cell size between 0-week training compared to their sedentary control (178.90 µm^2^ ± 33.08 vs 175.10 µm^2^ ± 27.86, p = ns) as well as in the CMs number-size proportion. Most CMs from both groups at this time point are almost identically located in the 101–200 mm^2^ interval. On the contrary, we found a statistically significant difference in mean average CMs surface area between 8-week training and control groups (315.10 µm^2^ ± 32.94 and 173.50 µm^2^ ± 20.41, p < 0.001; Figures 3J–L). The shifting of CM number percentage to greater surface areas in the 8-week training group demonstrated cardiac hypertrophy.

The differential average cell size in µm^2^ quantification and the shifts in CM’s cross-sectional area among training and sedentary groups are illustrated for all training groups in Figures 3D–O. Figures 3M–O showed the larger mean CM surface area of 12-week training compared to the sedentary group (276.80 µm^2^ ± 23.37 vs 153.30 µm^2^ ± 20.90, p < 0.001). The shift of CM size towards the larger surface area (201–300 mm^2^) was also observed in the 8- and 12-week training groups. Therefore, we analyzed if physiological hypertrophy as a response to forced endurance exercise is accompanied by pathological hallmarks such as inflammation and/or fibrosis. Heart sections stained with hematoxylin-eosin did not visualize inflammatory cell infiltration, and picrosirius red did not identify fibrotic areas, nor was there a change in fibrosis gene expression (Supplementary Figures S1, S2).

In summary, our data suggest that cardiac hypertrophy in exercising animals became apparent starting from 8 weeks of forced training and reached a plateau. The maximum fold-change of CM surface area was recorded in the 8-week and 12-week training groups, which showed approximately 1.8-fold CM enlargement compared to the sedentary controls. Meanwhile, the 2- and 4-week training groups showed only 1.26 and 1.34-fold CM enlargement compared to their sedentary controls.

### 3.4 Forced exercise induces more profound cardiac gene expression than voluntary running

To better understand the mechanisms of exercise-induced cardiac remodeling, we performed bulk-RNA sequencing using sedentary controls and heart samples from 8 weeks of training. Transcriptional profiles of sedentary and training hearts were obtained using the Illumina NovaSeq^®^ 6000 next-generation sequencing system (Illumina, San Diego, USA). We compared our datasets of forced running with previously published datasets of 8-week voluntary running models to compare models and gain better insight into gene expression profiling (Lerchenmüller et al., 2022).

**FIGURE 4:**
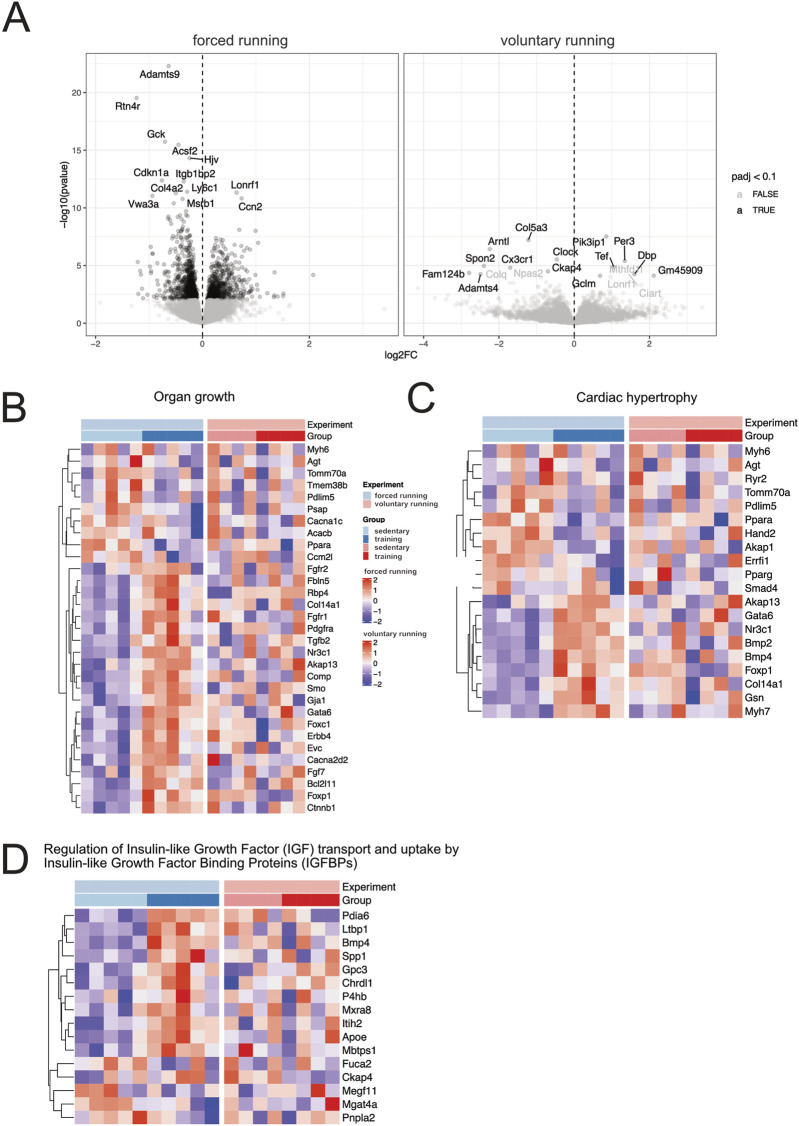
Gene expression analysis of forced and voluntary running models Volcano **(A)** and heat maps showing differentially expressed genes based on clustered genes annotated in several selected biological processes of organ growth **(B)**. Heat maps related to cardiac hypertrophy **(C)** and regulating IGF uptake and transport **(D)** are shown. Only genes considered significantly differentially expressed (False Discovery Rate (FDR) <10%) are included in the analysis.

Figure 4A depicts the volcano plot, which shows the average expression levels and extent of comparison between forced and voluntary running models. Surprisingly, the training effect on cardiac gene expression differed among forced vs voluntary running models.

Among the differentially expressed genes, several genes are involved in the cell cycle, cellular proliferation, and negative regulators of apoptosis (*Ccn2*, *Bcl2-like 1*, *Hspb6*, *Akap1*). Genes involved in glucose metabolisms, fatty acid homeostasis, transport, and storage, such as *Plin5, Fabp3, Gck*, and *Acsf2,* were downregulated after training, indicating a switch in metabolic substrate utilization. Genes involved in aerobic respiration, oxidative phosphorylation, and electron transport chain were downregulated in exercising mice compared to the sedentary control, indicating a shift in the CM metabolic requirements. Lastly, genes that participate in the collagen biosynthesis process, namely, *Col4a2* and *Adamst9,* were also downregulated in the forced running model. Interestingly, compared to the 8-week voluntary running, the effect of training was more profound after forced running.

We further investigated hypertrophy gene hallmarks in the training animals by selecting differentially expressed genes in critical biological processes such as organ growth (Figure 4B), cardiac hypertrophy (Figure 4C), the regulation of insulin-like growth factor (IGF) transport and uptake by the IGF binding proteins (Figure 4D). Essential genes involved in CM β-myosin heavy chain and protein synthesis, such as *Myh7, Foxp1*, and *Gata6,* were more profoundly upregulated after forced training.

**FIGURE 5:**
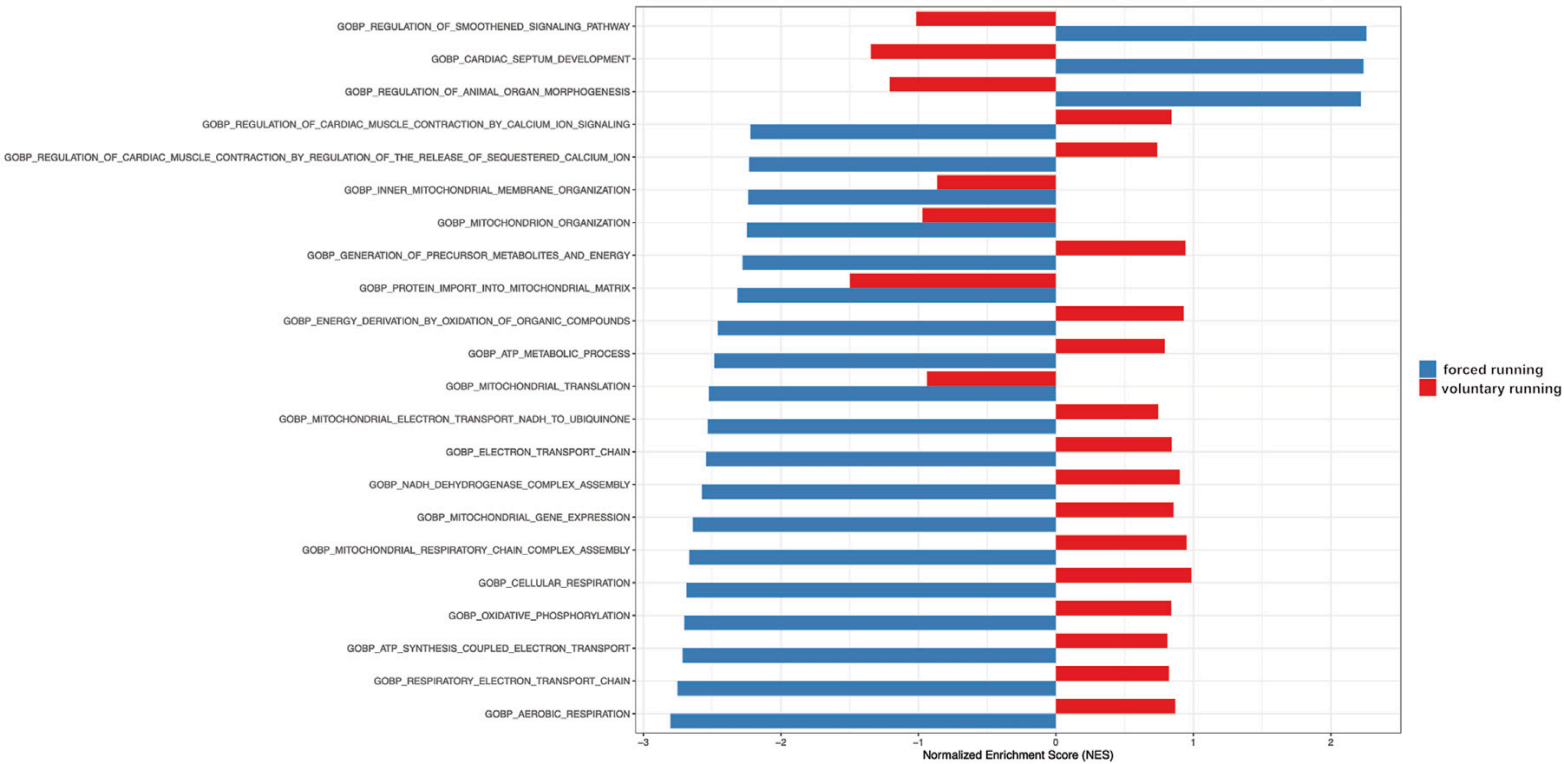
Gene Set Enrichment Analysis (GSEA) of gene ontology gene in forced vs voluntary running for 8 weeks. Gene Set Enrichment Analysis (GSEA) of gene ontology gene sets shows only the most relevant biological processes. The blue color indicates forced running. The red color shows voluntary running.

Figure 5 shows the gene set enrichment analysis (GSEA) showing relevant biological processes among forced vs voluntary running. Based on the GSEA analysis, most upregulated genes in the forced training regulate cardiac morphogenesis and development as well as mitochondrial and metabolism gene expression patterns.

In conclusion, after 8 weeks, treadmill exercise leads to a more profound alteration in gene expression in the hypertrophied heart compared to voluntary running.

## 4 Discussion

This study aimed to evaluate forced treadmill endurance exercise on the heart by assessing cardiac morphology, heart function, and transcriptomic profiling in mice. Gene expression after induction of exercise-induced cardiac remodeling was compared to a voluntary running model to give new insights in both mouse exercise models.

Previous studies used several animal species, including rodents, swine, dogs, and rabbits, and different training models to observe alterations in cardiac phenotypes (Wang et al., 2010; Polyák et al., 2018). Training modalities varied from voluntary wheel running, swimming, and forced training on the treadmill to sled pulling in large animals. Parameters used to investigate the occurrence of cardiac hypertrophy were often limited only to morphometric characterization, such as heart and body weight ratio, left or right ventricular mass, and CM dimension measurement (Wisløff et al., 2001; Diffee and Nagle, 2003).

Our study shows that intensity-controlled forced endurance training in mice induces morphological cardiac hypertrophy beginning at 8 weeks of exercise. The training animals had lower body weights, increased heart weights, and heart weight/tibia length ratio. Tibia length was used as a denominator in our study due to body weight changes over training duration. Using immunofluorescence staining, we show that significantly more CM in 8- and 12-training groups shifted towards larger cross-sectional surface areas, indicating cardiac hypertrophy.

In our study, we performed a rather rigorous training protocol with maximum training of 60–120 min daily for 5 days/week. Several studies depict various training protocols in rats and mice treadmill training programs. We adopted our protocol to the published protocol of Kemi et al. (Kemi et al., 2002), additionally including other time points that could potentially be used to save workload and resources.

A limitation of the study is that forced training models in animals potentially bear underlying confounders such as stress induction and circadian disruption, which cannot be entirely excluded by our experimental setting and make its one-to-one translation into human endurance training across species difficult. It remains elusive whether forced vs voluntary training in humans also depicts differences in cardiac or skeletal gene expressions.

Strict stress monitoring was performed according to the animal score sheet approved by the local authorities, including (general health, body conditions score, activity, self-care condition, fecal and urin status, respiratory/circulation status). However, none of the mice analysed reached a score to be excluded from the running protocol.

We subsequently investigated the cardiac function and volume during training using a dedicated small animal PET/CT scan. EF did not alter across different groups at various time points, underlining that cardiac volumes did not change although observable exercise-induced cardiac remodeling. Furthermore, cardiac remodeling is highly regulated at the molecular level, involving several biological organ growth and development processes. Therefore, we conducted transcriptomic analyses of the control and hypertrophied hearts to show relevant processes and molecular pathways. In bulk RNA sequencing analysis in 8 weeks of training animals, the most differentially expressed genes between forced training and control animals were involved in regulating cell cycle and apoptosis, indicating that they might play a role in the development of hypertrophy. Several genes related to increasing sarcomere β-myosin heavy chain and other regulatory mitogen-activated protein kinases (MAPK) pathway, anchoring proteins synthesis, and glucose and lipid homeostasis were upregulated after induced cardiac hypertrophy.

Furthermore, signaling pathways involved in organ remodeling, such as *FGF, PDGF, TGF-β/BMP/SMAD*, and genes involved in cardiac morphogenesis and development, were found to be reactivated in response to training. In addition, the biological processes involved in aerobic respiration, oxidative phosphorylation, and electron transport chain were decreased after training, according to our GSEA results. These study results serve as a baseline for further research and await further investigations.

Several pathways related to organ growth and cardiac hypertrophy were differentially regulated (see Figure 4). However, discussion of all relevant pathways is beyond the scope of this manuscript. Here, we want to focus on the regulation of Insulin-like Growth transport and uptake by Insulin-like growth factor binding proteins. In this analysis, differential gene expression comparing the forced vs voluntary running is evident. The review of Bass-Stringer and colleagues discusses the impact of insulin-like growth factor 1 (IGF1) phosphoinositide 3-kinase (PI3K) pathway as a critical pathway for mediating exercise-induced heart growth and modulating activity of the IGF1 PI3K pathway as an approach to mimic the protective effects of exercise on the heart (Bass-Stringer et al., 2021). Our data suggest that the forced running protocol could have benefits regarding this pathway compared to voluntary running models in a murine model. Interestingly, IGF1 has been demonstrated to be increased in elite athletes (soccer players) with enlarged hearts (Neri Serneri et al., 2001). The measurement of IGF1 was performed by the artery to coronary sinus concentration gradient during invasive electrophysiological studies. The data derived from humans is consistent with our mouse model and cardiac sequencing results, while IGF1 is released from cardiomyocytes. IGF1 binds to its plasma membrane IGF1 receptor and thereby initiates the activation of pro-hypertrophic signaling pathways (PI3K-protein kinase B (Akt) cascade), thereby leading to hypertrophy.

Remarkably, our data suggest that the alteration in gene expression is more prominent in forced vs voluntary running protocol. These results underscore the differences in forced vs voluntary running models in murine research.

Our data suggest that there is a difference in gene expression when forced or voluntary training is applied in mice. Our experiments show that forced (or intensity-controlled) might provide a stronger effect, at least regarding gene expression (especially regarding IGF1). Whether these results can easily be transferred to the debate of endurance training in humans is hard to tell due to the difference in species. RNA sequencing of cardiac or skeletal tissue of athletes is not feasible due to legal rules and ethical concerns. Further studies, such as RNA profiling in humans using blood samples, could potentially provide more insight into this ongoing discussion.

Our results showed that forced endurance training-induced body weight loss in exercising animals could be noticed early on. The difference in final body weight during the 12 weeks was recorded as 2.66 g less in the training group compared to sedentary controls (p < 0.001). The results are expected and consistent with previous studies, which showed that moderate-as well as high-intensity endurance training was effective in reducing total body weight by depleting the central fat accumulation of obese rodents, reflecting a better glycemic metabolic control as well as fatty acid metabolism (de Lade et al., 2018; Martin-Smith et al., 2020).

We show that exercise-induced cardiac remodeling can be observed morphometrically starting from 8 weeks of 2 hours per day intensity-controlled forced endurance training at a speed of 15 m/min. This result adds new insight compared to an experiment using the 129 SvJ/C57BL6 animal genetic background using a shorter training protocol (40 min/day) with higher speed (24 m/min), which failed to detect a change in heart weight/body weight ratio after 8 weeks duration (Han, 2013). This finding could be attributed to using body weight as the denominator of heart weight and could underline the importance of genetic background in mice. Several studies showed that forced middle-intensity endurance training by swimming 3 h/per day in rats increased heart weight by 30% after 180 h of training. Ninety minutes of swimming with a 2% total body weight load, three times/day for 7 weeks, also showed exercise-induced cardiac remodeling without signs of scar tissue formation (Dos Santos Soares et al., 2019). Another study protocol concluded that higher intensity endurance training on a treadmill in rats with a speed of 30 m/min, 30 min/day, 5 days/week for 8 weeks has also successfully induced cardiac remodeling characterized by increasing heart weight in training groups compared to sedentary control (Gunadi et al., 2019). Furthermore, higher-intensity continuous treadmill exercise in rodents (speed of 26.8 m/min, 10^o^ inclination, for 60 min, 5 days/week for 8 weeks) showed CM injury, increased cardiac troponin I, fibrosis, and expressions of genes involved in the development of pathological hypertrophy (Yan et al., 2021). However, not performing endurance training continuously could reverse these potential adverse effects. Recent studies showed that both in animal and human studies, high-intensity interval and continuous moderate-intensity endurance training can evoke beneficial effects in glucose metabolism, lipolysis, and aerobic capacity. Furthermore, cardiorespiratory fitness augmentation by exercise-induced cardiac remodeling reduces sarcopenia and prevents neurocognitive deterioration in aging individuals (Chavanelle et al., 2017; Seldeen et al., 2018; Martin-Smith et al., 2020; Tsai et al., 2021). A moderate-high intensity aerobic exercise using a treadmill in rodents for 60 min/day, 5 days/week for 8–12 weeks led to 17%–32% augmentation of CM size by activating concurrently several of these pathways, promoted neo-angiogenesis and altered cardiac expression of both Bcl-2 and heat shock protein 70 as markers of apoptosis inhibition (Kemi et al., 2002). A meta-analysis showed that using treadmill training protocols in mice of a total 5–8 weeks duration, irrespective of training intensity, achieved the desired outcomes compared to shorter (<4 weeks) or longer (>8 weeks) exercise duration. Failure of training adaptation was expected in a shorter duration. Training duration of fewer than 60 min/day was also expected to affect exercise-induced biochemical alterations [40] substantially. Our study indicates that heart weight/tibia length ratio and CM surface area indicators for cardiac hypertrophy were optimally quantified in the 8-week training group, which runs for 120 min/day, 5 days weekly. A similar effect was found in our 12-week training group. The reduced sample size in ther 12-week training group may limit the statistical power, which is limitation. The data suggest a strong trend, which is in line with the cardiac hypertrophy in the 8-week training cohort.

Experimental forced endurance training using a treadmill machine might generally induce stress in the animals due to stimuli. The training was conducted during the day, which may impact their natural circadian rhythm and behavior, thus building stress responses (Svensson et al., 2016). A free-access voluntary running wheel experiment could be an alternative to forced treadmill training protocols in evaluating exercise-induced cardiac remodeling without exposing additional stress stimuli in training animals (Wang et al., 2010). However, some studies concluded that voluntary wheel training in mice failed to alter CM even after more extended training periods (Natali et al., 2001; Eisele et al., 2008). We examined whether features of pathological hypertrophy have occurred after 8 weeks of training. Pathological versus training-induced cardiac remodeling is characterized by disorganization of cardiac structure, inflammatory cell infiltration, and fibrosis (McMullen and Jennings, 2007). In addition to WGA, we performed hematoxylin-eosin and picrosirius red staining to detect pathological features.

Nevertheless, we found neither sign of inflammation nor fibrosis in all training hearts, irrespective of training duration. These results align with the recent outcomes showing that endurance training (duration varied from 28 days to 35 months) downregulated inflammatory cytokines and fibrosis markers in the blood tissues, as well as molecular expression levels in chemotherapy-damaged and aging hearts (Wright et al., 2014; Yang et al., 2020). This result underlined that cardiac remodeling induced by endurance training exhibits no signs of pathological hallmarks.

The ECG-gated reconstructed PET/CT image acquisitions were processed to evaluate LV volumes in systole and diastole. The EF and CO can be derived by assessing EDV, ESV, SV, and heart rate/minute. Eccentric cardiac hypertrophy, which usually occurs in triathlon athletes after roughly 2 years of training, is characterized by a significant increase in EDV and SV with relatively maintained ESV and LVEF (Scharf et al., 2010). On the other hand, pathological concentric hypertrophy, often combined with eccentric chamber dilatation in failing hearts, created lower SV and reduced LVEF with relatively larger EDV and ESV (Harbo et al., 2020). Physiological hypertrophy can compensate for the lower baseline heart rate with increased SV to maintain the equation of relatively normal CO, indicating a prevailing overall cardiac function (Carter et al., 2003). The training mice had stable EDV, ESV, SV, and LVEF with neither sign of heart chamber dilatation nor failing CO.

The RNA sequencing analysis showed that 8-week training hearts highly upregulated genes involved in cell cycle, apoptosis, fatty acid homeostasis, organ growth pathways, and cardiac developmental processes. To gain new insight into the forced running model, we used a previously published dataset on exercise-induced cardiac remodeling induced by 8-weeks of voluntary running (Lerchenmüller et al., 2022). In the Lerchemüller publication, aside from the older mice, they used younger mice aged 10 to 12- weeks and performed 8 weeks of voluntary running to induce exercise-induced cardiac remodeling.

Several essential genes that inhibit apoptosis and checkpoint for cell cycle progression toward mitosis, such as *Bcl2l1*, *Ccn2*, and *Cdkn1a,* were downregulated in forced training hearts. Interestingly, Cdkn1 is the inhibitor of *Cdk1*. Overexpression of Cdk1 in CM-induced cell division in post-mitotic mice, rats, and human CM (Mohamed et al., 2018). However, the role of the downregulated translated protein and downstream pathways in promoting adult CM reentry to the cell cycle or even enhancing cell survival remains to be discovered.

Mice performing voluntary running wheel for 3 weeks have been shown to downregulate cell cycle inhibitors and apoptosis regulators (*P16, P53*, and *Cell-cycle-checkpoint-kinase 2*) in vascular endothelial cells (Werner et al., 2009). Furthermore, a previous publication has shown that the microRNAs in endurance training athletes might have a pivotal role in modulating downstream effects of cell cycle proteins cyclin-dependent kinases in skeletal muscle biopsies (Nielsen et al., 2010). However, there is a limited number of previous publications in basic science research that can connect the alteration in CM cell cycle regulator protein expression in response to endurance training. Previous studies managed to show that endurance training of 4–8 weeks in rodents by voluntary wheel running might be responsible for enhancing the cardiomyogenesis assessed using ^15^N-Thymidine multi-isotope imaging mass spectrometry and increasing the number of resident endogenous cardiac stem cell progenitors (Vujic et al., 2018; Lerchenmüller et al., 2022). Interestingly, swimming exercise also increased c-Kit cardiac stem cells in physiological hypertrophied hearts (Leite et al., 2015). Technological advances are needed due to the limitation of reliable methods to monitor CM proliferation and regeneration activities, especially *in vivo* (Walsh et al., 2010).

Several genes in response to endurance training were upregulated in our RNA sequencing analysis. The A-kinase anchoring proteins (AKAP) are essential scaffolding proteins involved in subcellular MAPK signal transduction cascades leading to cardiac hypertrophy. For example, previous studies showed that deficiency of AKAP in mice exhibited an accelerated progression to heart failure, increased collagen deposition apoptosis, and attenuated cardiac hypertrophy after angiotensin II administration and transverse aortic ligation. Recent genome-wide expression analysis in mice indicated the essential role of *in vivo* AKAP13-PKD1 signaling circuits in regulating CM contraction, apoptosis, and energy substrate metabolism during the physiological response of cardiac hypertrophy (Taglieri et al., 2014; Johnson et al., 2015). The *Akap13* gene was upregulated in 8 weeks of forced training animals, confirming the importance of these proteins in exercise-induced cardiac remodeling.

*Myh6* and *Myh7* express sarcomeric contractile proteins α- and β-myosin heavy chains, which are significantly upregulated in the cardiac remodeling. Interestingly, the Myh7 expression, as an example, was more robust in the forced vs voluntary running model (Figure 4C; Supplementary Figure S3). Increased expression of α-myosin heavy chains by Myh6 is associated with improved left ventricular contractility in healthy adults who performed high-intensity interval training. The balance between these two isoform proteins maintains proper physiologic cardiac contraction. Reexpression of embryonal β-myosin heavy chain in adult hearts is associated with cardiac hypertrophy in response to muscle injury and remodeling (Hsieh et al., 2022). Our results suggest that Myh7 is upregulated in forced training hearts, while other markers of fetal gene activation (e.g., ANP and BNP) did not change, arguing against the re-activation of fetal programs (Kuwahara et al., 2012). Reactivation of fetal gene expression is attributed to pathological remodelling. However, in this study, there was no evidence of cardiac malfunction, inflammation, or fibrosis at least on the functional analysis and gross histology. However, it remains possible that hidden stressors beyond the scope of our analysis could contribute to gene expression alterations in forced vs voluntary running models.

Comparing the gene expression of independent data sets might be influenced by a batch effect, consisting of technical and experimental aspects.

Moreover, several studies indicated that experimental exercise training on cardiovascular functions in larger animals such as dogs, rabbits, and swine was more favorable due to the resemblance of anatomy and structure to the human heart and vasculature (Polyák et al., 2018; Tharp et al., 2019). Nevertheless, ethical clearance, limitations in genetic manipulation, and higher costs are the major obstacles to performing such studies. Furthermore, the interpretation of the results should be translated carefully due to fundamental differences and limitations (Rai et al., 2017).

This study presents the morphometric, histological, and transcriptomic alterations in exercise-induced cardiac remodeling due to forced intensity-controlled endurance training on a small-animal treadmill compared to a voluntary running model. The phenotypic changes were subsequently confirmed using immunofluorescence to visualize the CM size change by the cross-sectional area without detecting inflammatory or fibrotic tissue remodeling. The trained hearts maintain a normal cardiac function despite an exercise-induced cardiac phenotype. Here, we provide data comparing the two commonly used exercise models for cardiac remodeling: forced vs voluntary running. These comparisons show interesting effects on transcriptomic levels regarding gene expression analysis in cellular proliferation, apoptosis, contractile protein synthesis, glucose transport, organ growth, and cardiac hypertrophy. Thus, this work provides new insights into the 2 mouse models of exercise-induced cardiac remodeling and should help researchers choose the most suitable exercise model for their specific objective.

## Data Availability

The original contributions presented in the study are publicly available. This data can be found here: https://www.ncbi.nlm.nih.gov/sra/?term=PRJNA811435 and in the GEO repository under accession number GSE308626.

## Abbreviations

^18^F-FDG, 2-deoxy-2-[^18^F]fluoro-D-glucose; CM, Cardiomyocyte; ECG, Electrocardiogram; EDV, End-diastolic volume; EF, Ejection fraction; ESV, End-systolic volume; PET/CT, Positron emission tomography/computed tomography; SV, Stroke volume.
