# Supplementary material for: Forced intensity-controlled endurance training on a small-animal treadmill machine inducing murine cardiac hypertrophy: insights and comparison to voluntary running models
Source: Front Pharmacol. 2025 Oct 9;16:1682751. doi: 10.3389/fphar.2025.1682751 (PMC12545138; doi:10.3389/fphar.2025.1682751)
Supplement: Supplementary file 1 [file Supplementaryfile1.docx]

Forced intensity-controlled endurance training on a small-animal treadmill machine inducing murine cardiac hypertrophy: insights and comparison to voluntary running models

Maximillian Fischer ^1,2 *^, Agus Simahendra ^1^, Tobias Straub ^3^, Stefan Brunner ^1,2^, , Bartolo Ferraro ^1,2,4,†^, Ludwig T. Weckbach ^1,2,4,†^

^1^Department of Medicine I, LMU University Hospital, LMU Munich, Marchioninistraße 15, 81377 Munich, Germany.

^2^DZHK (German Center for Cardiovascular Research), partner site Munich Heart Alliance, Munich, Germany.

^3^Core Facility Bioinformatics, Biomedical Center, Ludwig-Maximilians-University Munich, Planegg-Martinsried, 82152 Munich, Germany

^4^Biomedical Center, Institute of Cardiovascular Physiology and Pathophysiology, Ludwig-Maximilians-University Munich, Planegg-Martinsried, 82152 Munich, Germany

*** Correspondence:**Dr. Maximilian Fischer
[maximilian.fischer@med.uni-muenchen.de](mailto:maximilian.fischer@med.uni-muenchen.de)

**† These authors contributed equally to this work.**

Keywords: cardiomyocytes, physiological cardiac hypertrophy, endurance training, small-animal treadmill

# Supplementary Figure

**Supplementary Figure S1:**Representative heart images stained with hematoxylin-eosin (left side) and picrosirius red (right side) to show absent inflammation and fibrosis respectively. Fibrosis quantification of cumulative 0-week, 8-week and 12-weeks hearts on the right. On each staining section, the sedentary controls were always pictured on the left and their training counterparts on the right **(A)** Baseline 0-week hematoxylin-eosin, **(B)** 0-week picrosirius red, **(C)** 8-weeks hematoxylin-eosin, **(D)** 8-weeks picrosirius red, **(E)** 12-weeks hematoxylin-eosin, **(F)** 12-weeks picrosirius red. N = 10 mice per group. Bar length represents 1000 µm.

**Supplementary Figure S2:**Gene expression analysis of fibrosis genes comparing sedentary and forced running.

**Supplementary Figure S3:**Gene expression analysis of fetal gene reactivation, including ANP (Nppa), BNP (Nppb), Myh7, and Myh6 in forced vs. voluntary running.
